# IL-10-mediated signals act as a switch for lymphoproliferation in Human T-cell leukemia virus type-1 infection by activating the STAT3 and IRF4 pathways

**DOI:** 10.1371/journal.ppat.1006597

**Published:** 2017-09-14

**Authors:** Leila Sawada, Yoshiko Nagano, Atsuhiko Hasegawa, Hikari Kanai, Kai Nogami, Sayaka Ito, Tomoo Sato, Yoshihisa Yamano, Yuetsu Tanaka, Takao Masuda, Mari Kannagi

**Affiliations:** 1 Department of Immunotherapeutics, Tokyo Medical and Dental University, Graduate School of Medical and Dental Sciences, Bunkyo-ku, Tokyo, Japan; 2 Department of Medical Technology, School of Health Sciences, Tokyo University of Technology, Ota-ku, Tokyo, Japan; 3 Department of Rare Disease Research, Institute of Medical Science, St. Marianna University School of Medicine, Kawasaki, Kanagawa, Japan; 4 Department of Immunology, Graduate school of Medicine, University of the Ryukyus, Nishihara-cho, Okinawa, Japan; University of Illinois at Chicago College of Medicine, UNITED STATES

## Abstract

Human T-cell leukemia virus type-1 (HTLV-1) causes two distinct diseases, adult T-cell leukemia/lymphoma (ATL) and HTLV-1-associated myelopathy/tropical spastic paraparesis (HAM/TSP). Since there are no disease-specific differences among HTLV-1 strains, the etiological mechanisms separating these respective lymphoproliferative and inflammatory diseases are not well understood. In this study, by using IL-2-dependent HTLV-1-infected T-cell lines (ILTs) established from patients with ATL and HAM/TSP, we demonstrate that the anti-inflammatory cytokine IL-10 and its downstream signals potentially act as a switch for proliferation in HTLV-1-infected cells. Among six ILTs used, ILTs derived from all three ATL patients grew much faster than those from three HAM/TSP patients. Although most of the ILTs tested produced IFN-γ and IL-6, the production of IL-10 was preferentially observed in the rapid-growing ILTs. Interestingly, treatment with exogenous IL-10 markedly enhanced proliferation of the slow-growing HAM/TSP-derived ILTs. The IL-10-mediated proliferation of these ILTs was associated with phosphorylation of STAT3 and induction of survivin and IRF4, all of which are characteristics of ATL cells. Knockdown of STAT3 reduced expression of IL-10, implying a positive-feedback regulation between STAT3 and IL-10. STAT3 knockdown also reduced survivin and IRF4 in the IL-10- producing or IL-10- treated ILTs. IRF4 knockdown further suppressed survivin expression and the cell growth in these ILTs. These findings indicate that the IL-10-mediated signals promote cell proliferation in HTLV-1-infected cells through the STAT3 and IRF4 pathways. Our results imply that, although HTLV-1 infection alone may not be sufficient for cell proliferation, IL-10 and its signaling pathways within the infected cell itself and/or its surrounding microenvironment may play a critical role in pushing HTLV-1-infected cells towards proliferation at the early stages of HTLV-1 leukemogenesis. This study provides useful information for understanding of disease mechanisms and disease-prophylactic strategies in HTLV-1 infection.

## Author summary

It has been a long-unsolved question why HTLV-1 can cause totally different diseases such as ATL and HAM/TSP, manifesting as malignant lymphoproliferation and chronic inflammation, respectively, without disease-specific viral differences. Although the constitutive NF-κB activation in HTLV-1-infected cells has been implicated for HTLV-1 pathogenesis, NF-κB potentially contributes to both leukemogenesis and inflammation. Here, we report that IL-10 and its downstream STAT3 pathway play a critical role in proliferation of HTLV-1-infected cells. Our finding that IL-10 converted the nature of the poorly proliferative HTLV-1-infected cells from HAM/TSP patients into rapidly-growing cells resembling characteristics of ATL cells implies the importance of the host IL-10-dominant microenvironment in building the proliferative machinery in HTLV-1-infected cells. The positive-feedback regulation between IL-10 and STAT3 would likely accelerate this process. These findings emphasize the importance of the STAT3 pathway in addition to the NF-κB pathway in the process leading to leukemogenesis in HTLV-1 infection. This study provides evidence that the host microenvironment is potentially involved in the disease mechanism, partly explaining the reason for the different disease outcomes in HTLV-1 infection.

## Introduction

Human T-cell leukemia virus type-1 (HTLV-1) is a retrovirus that infects approximately 5–10 million people worldwide [1]. Although most infected individuals remain asymptomatic, approximately 4% develop adult T-cell leukemia/lymphoma (ATL) and less than 2% develop HTLV-1-associated myelopathy/tropical spastic paraparesis (HAM/TSP) [2–4]. ATL is an aggressive lymphoproliferative disease with severe immunosuppression [5], while HAM/TSP is a chronic inflammatory disease of the spinal cord featuring progressive demyelination [6, 7]. The reasons why the same virus is the causative agent of two vastly different diseases are unknown. Previous reports have demonstrated that there are no disease-specific HTLV-1 strains causing either ATL or HAM/TSP [8, 9], indicating that the different clinical outcomes of infection could be attributed to host factors. However, the essential host factors and the mechanisms regulating the initiation of lymphoproliferative or inflammatory disease in HTLV-1 infection remain unclear.

Thus far, several differences in host factors have been reported between the two diseases. For example, the incidence of disease is greater in males with ATL [10], but greater in females with HAM/TSP [4]. Furthermore, the association of mother-to-child transmission of HTLV-1 infection with ATL development has also been suggested [11]. Analysis of genetic background indicated that the frequencies of several HLA alleles might differ in HTLV-1 infection-associated diseases [12, 13]. The strength of the HTLV-1 Tax-specific cytotoxic T lymphocyte (CTL) response also differs between the two diseases, being elevated in HAM/TSP patients but impaired in ATL patients [14, 15]. Even though HTLV-1-specific CTLs were detected in ATL patients, they usually do not sufficiently expand in response to their cognate antigens, suggesting the existence of an immunosuppressive mechanism [16, 17].

Another difference between the different clinical outcomes observed in HTLV-1 infection is the virus expression level. HTLV-1 gene expression is usually low *in vivo*; undetectable at the protein level and barely detectable at the mRNA level [18, 19]. HAM/TSP patients have slightly higher HTLV-1 mRNA levels, when compared with asymptomatic HTLV-1 carriers or ATL patients [20]. Viral protein expression is an important aspect of HTLV-1 infection. The pleiotropic HTLV-1 protein, Tax, can induce genes related to cell survival and proliferation, as well as inflammatory responses [21–23], likely contributing to viral pathogenesis. Additionally, Tax protein is a dominant target antigen for CTL responses [15, 24]. Enhanced expression of Tax may partly explain the elevated Tax-specific CTL responses observed in HAM/TSP patients. HTLV-1 basic leucine zipper factor (HBZ) encoded by the antisense HTLV-1 genome is also a multifunctional protein implicated for HTLV-1 pathogenesis [25, 26]. However, the expression levels of HBZ do not differ among the diseases [27]. Recently, we reported that stromal cells can inhibit HTLV-1 expression via type I interferon (IFN) responses [28], suggesting that the innate immune response might be involved in the difference in HTLV-1 expression levels between diseases.

The innate host immune response itself may also contribute to HTLV-1 pathogenesis. HTLV-1 infected T-cells exhibit constitutive activation of NF-κB, a critical transcription factor for both leukemogenesis and inflammation [23, 29, 30]. Although Tax possibly activates NF-κB, it cannot fully explain the mechanism of constitutive NF-κB activation, because NF-κB is also activated in Tax-negative ATL cells [29]. The presence of genetic alterations in the mediators of T-cell receptor signaling pathway in ATL cells may partly be involved in the mechanisms of NF-κB activation [31]. In addition, our recent study indicated that ATL cells constitutively express antisense RNA including the HTLV-1 LTR region, resulting in constitutive activation of NF-κB through activation of the double-stranded RNA (dsRNA)-dependent protein kinase (PKR) [32]. This provided a new angle to understanding how HTLV-1 produces constitutive NF-κB activation without detectable Tax protein, once more indicating a link between HTLV-1 pathogenesis and innate host anti-viral immune responses.

The potential involvement of host anti-viral innate responses in HTLV-1 pathogenesis leads us to hypothesize that individual differences in the host response might play a role in the clinical outcomes of HTLV-1-infection. Few studies have focused on the difference in innate immune responses between HAM/TSP and ATL patients. Several reports have demonstrated differences in the serum cytokine profiles of HTLV-1-infected individuals with various diseases. Among them, it is noteworthy that ATL patients present with higher levels of IL-10 compared with asymptomatic HTLV-1 carriers and healthy donors [22, 33], while this difference is not significant between HAM/TSP patients and asymptomatic HTLV-1 carriers [34, 35]. IL-10 is a pleiotropic cytokine with immunoregulatory and anti-inflammatory functions, the expression of which is driven mainly by NF-κB and AP-1 following stimulation of various immune receptors including TLRs and PKR [36–38].

In the present study, to investigate host factors regulating HTLV-1-mediated lymphoproliferative and inflammatory diseases, we established IL-2-dependent HTLV-1-infected T-cell lines (ILTs) from patients with ATL and HAM/TSP, and characterized their cytokine production, viral protein expression, and proliferative abilities. We demonstrate that ATL-derived ILTs grew faster than HAM/TSP-derived ILTs, which was associated with their ability to produce IL-10. We further demonstrated that exogenously added IL-10 converted the HAM/TSP-derived ILT cells into cells with better growth capabilities, which was associated with induction of survivin and IFN regulatory factor 4 (IRF4), resembling ATL cells. This study provides evidence that the IL-10-mediated signal is critical for determining the process that HTLV-1-infected cells gain proliferative abilities in the early stages toward HTLV-1 leukemogenesis.

## Results

### IL-10 promotes proliferation of IL-2-dependent HTLV-1-infected T-cell lines (ILTs)

**Fig 1:**
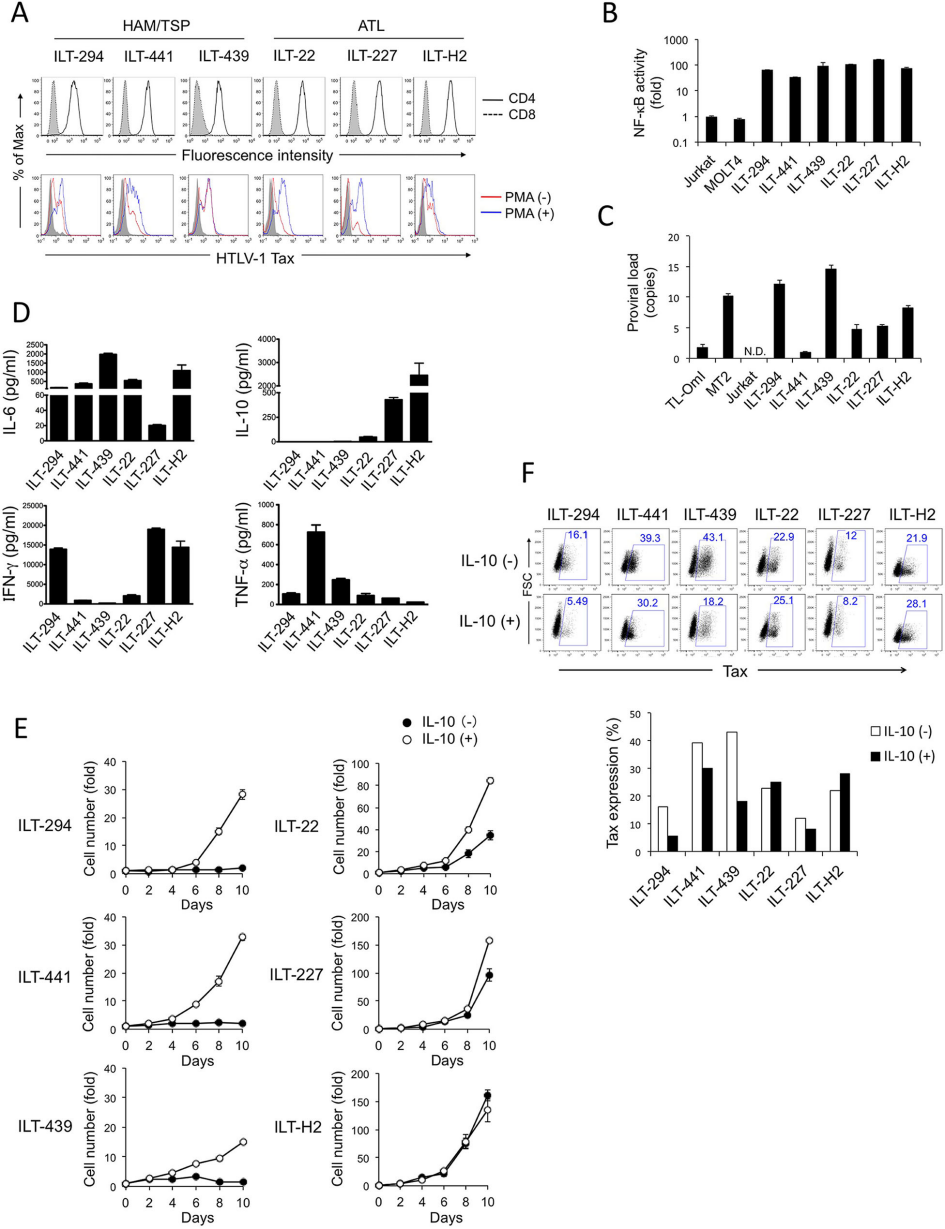
IL-10 promoted cell growth of IL-2-dependent HTLV-1-infected T-cell lines (ILTs). ILTs were established by long-term culture of PBMCs from patients with ATL (#22, #227, and H2) and HAM/TSP (#294, #439, and #441) in the presence of rhIL-2. **A.** Cell surface expression of CD4 (solid line) and CD8 (dotted line) (top), and intracellular expression of HTLV-1 Tax (bottom) with (blue) or without (red) stimulation with 50 ng/ml PMA for 18 h were analyzed by flow cytometry. Closed histograms indicate staining with control antibodies. **B.** NF-κB activities in ILTs were measured by a transient reporter assay. The luciferase activities were standardized by TK-RL activities and indicated as relative values against Jurkat. **C.** HTLV-1 proviral loads in ILTs were measured by qPCR. The results were standardized against TL-OmI (1.8 copies/cell) [78]. N.D., not detected. **D.** The cytokine profiles in the supernatants of ILTs were analyzed with a multiplex bead-based immunoassay, following incubation at a cell concentration of 10^6^/ml for 24 h in the presence of rhIL-2. The results are presented as the means and SD of duplicate samples. **E.** The growth of ILT cells in the presence (○) or absence (●) of rhIL-10 (20 ng/mL) was assessed by the trypan blue exclusion method. Cell cultures were diluted with fresh medium every 2 days, regardless of the cell growth, and the viable cell number was counted. The total live cell numbers in culture are presented as relative values compared with the cell number at day 0. All culture medium contained rhIL-2. **F.** Intracellular Tax expression in ILT cells cultured with or without rhIL-10 for at least 7 days, with the medium being replaced 2 days before harvest, was analyzed by flow cytometry, and the proportion of Tax-expressing cells (%) is indicated in the bar graph. In **A, B,** and **D**, HAM/TSP-derived ILT cells were cultured in the medium without rhIL-10 at least for 1 week before use. Similar results were obtained in two independent experiments.

ILTs were established by long-term culture of peripheral blood mononuclear cells (PBMCs) isolated from patients with ATL (#22, #227, and H2) and HAM/TSP (#294, #441, #439) in the presence of recombinant human IL-2 (rhIL-2). ATL-derived ILTs grew well in the presence of IL-2, while the growth of HAM/TSP-derived ILTs was much slower. All ILTs were CD4^+^ CD8^-^ (Fig 1A top panel). In ILT cells, Tax was usually detectable only in a small subpopulation, although all the ILT cells were infected with HTLV-1, as they expressed Tax after stimulation with PMA (Fig 1A bottom panel). In a reporter assay, elevated levels of NF-κB activities were detected in all ILTs tested, when compared with Jurkat or MOLT4 cells (Fig 1B). Tax expression levels and HTLV-1 proviral loads varied among ILTs regardless of their growth characteristics (Fig 1A and 1C). The ILTs also expressed different combinations of cytokines, as evaluated by a bead-based cytokine assay (Fig 1D). Notably, ATL-derived ILTs produced varying levels of IL-10, while HAM/TSP-derived ILTs expressed negligible levels of IL-10. Among the ILTs tested, ILT-H2 was the highest IL-10 producer. Most of the ILTs tested produced IL-6, although ILT-227 produced considerably less. ILT-441 had the greatest TNF-α production, and ILT-227, -H2 and -294 produced higher levels of IFN-γ than the others. Despite the preference of IL-10 production in some ILTs, there was no preference in GATA3 expression. ILTs expressed both *TBX21 (T-bet)* and *GATA3*, but lower levels of *RORC* and *FOXP3* mRNA, when compared with activated PBMCs from a healthy individual (S1 Fig).

Since ILTs producing higher levels of IL-10 appear to have increased proliferative capacity, we examined the significance of IL-10 on the proliferation of ILTs by adding rhIL-10 to the cell culture medium. Results demonstrated that, in all HAM/TSP-derived ILTs tested, the expansion of the cells was remarkably greater in the presence of rhIL-10 when compared with the cultures without rhIL-10 (Fig 1E). ATL-derived ILTs grew well in the absence of rhIL-10, and ILT-22 that had slightly slower growth ability than the other ATL-derived ILTs presented further growth enhancement in the presence of rhIL-10. Similar IL-10-mediated growth enhancement was observed in ILT-227 to a lesser degree, while ILT-H2 showed no difference or a slight decrease in cell growth following IL-10-treatment.

We next assessed the effects of IL-10-treatment on HTLV-1 Tax expression, as Tax has been shown to play important roles in the transformation of HTLV-1-infected cells [23]. In ILT-294 and ILT-439, Tax expression was clearly reduced in the presence of rhIL-10 (Fig 1F). In the other ILTs, the effects of IL-10 on Tax expression levels were limited and varied among experiments.

Thus, IL-10 production was associated with proliferation of ILTs, and exogenously added rhIL-10 promoted cell growth, especially in ILTs derived from HAM/TSP patients. However, this was not correlated with HTLV-1 Tax expression.

### IL-10 enhances expression of Ki67 and survivin in HAM/TSP-derived ILTs

**Fig 2:**
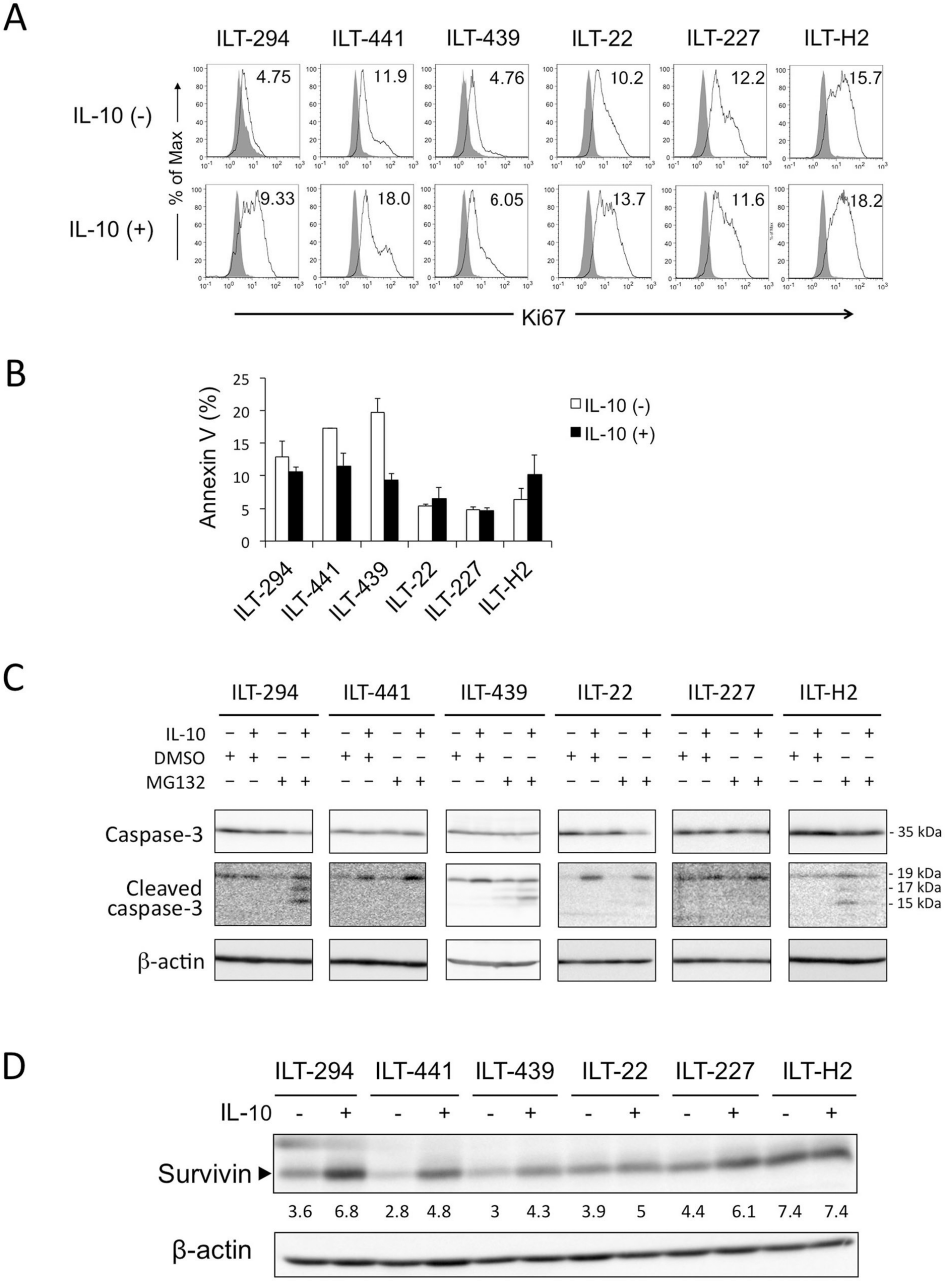
IL-10 enhanced expression of Ki67 and survivin in HAM/TSP-derived ILTs. ILTs were cultured with or without IL-10 for 7–11 days, and subjected to flow cytometry (A, B) and immunoblot assays (C, D). **A**. Permeabilized cells were stained with anti-Ki67 (solid line) or control (closed histogram) antibodies. Numbers indicate MFI of Ki-67 expression. **B**. Cells were stained with Annexin V, and the proportion of apoptotic cells (%) was indicated as the mean and SD of 2–3 independent experiments. The representative flow cytometry data are shown in S2 Fig. **C.** ILTs cultured with or without IL-10 were treated with MG132 (10 μM) for 3 h, then subjected to immunoblot assays probed with antibodies to caspase-3, cleaved caspase-3, and β-actin. Approximate sizes (kDa) of caspases are indicated. The results of a similar experiment without MG132-treatment is shown in S3 Fig. **D.** ILTs cultured with or without IL-10 were subjected to immunoblot assays to detect survivin. The number under each band represents the relative value of survivin expression normalized against β-actin. Similar results were obtained in two independent experiments.

Since IL-10 promoted the expansion of ILT cells (Fig 1E), we next investigated the effects of IL-10 on cell cycling and apoptosis. In the absence of exogenous rhIL-10, the levels of Ki67 expression in HAM/TSP-derived ILT cells were lower than those of ATL-derived ones, consistent with their growth characteristics. In the presence of rhIL-10, expression of Ki67 increased in most of the ILTs tested, indicating that IL-10 promoted progression of the cell cycle (Fig 2A). In the absence of rhIL-10, HAM/TSP-derived ILT cultures contained considerable proportions of apoptotic cells detected by Annexin V-staining, which were decreased in the presence of rhIL-10 (Fig 2B, S2 Fig). In contrast, ATL-derived ILTs contained lower proportions of apoptotic cells, which were slightly increased or not affected by rhIL-10. Interestingly, both HAM/TSP- and ATL-derived ILTs exhibited detectable levels of cleaved caspase-3 at 19 kDa in the absence of rhIL-10 in immunoblotting assays. The lower bands of cleaved caspase-3 (15–17 kDa) were also detectable in some samples treated with the proteasome inhibitor MG132, indicating the presence of active apoptotic signaling in these cells. Further increases in the 19 kDa cleaved caspase-3 were observed in ILT-294, ILT-441, ILT-439, ILT-22 and ILT-227 cells cultured in the presence of rhIL-10, especially in the samples treated with MG132 (Fig 2C, S3 Fig). In ILT-H2, which exhibited the highest levels of IL-10 production among ILTs tested, the effect of rhIL-10 on cleaved caspase-3 levels was limited and varied among experiments.

Since there was an apparent paradox that IL-10 suppressed apoptosis despite enhanced caspase-3 cleavage, especially in the HAM/TSP-derived ILTs tested, we next assessed the levels of survivin. Survivin is known to inhibit apoptosis by directly interacting with active caspases [39] and is expressed in ATL cells [40–42]. Immunoblotting analysis indicated that IL-10 enhanced survivin expression, especially in ILT-294, ILT-441, and ILT-439 cells (Fig 2D). Survivin expression in ILT-22, ILT-227 and ILT-H2 was slightly enhanced or unchanged following IL-10-treatment. These observations suggested that, although ILTs have constitutively active apoptotic machinery, IL-10 promoted cell cycle progression and inhibited apoptosis partly through the induction of survivin.

### IL-10 strongly activates STAT3, especially in HAM/TSP-derived ILT cells

**Fig 3:**
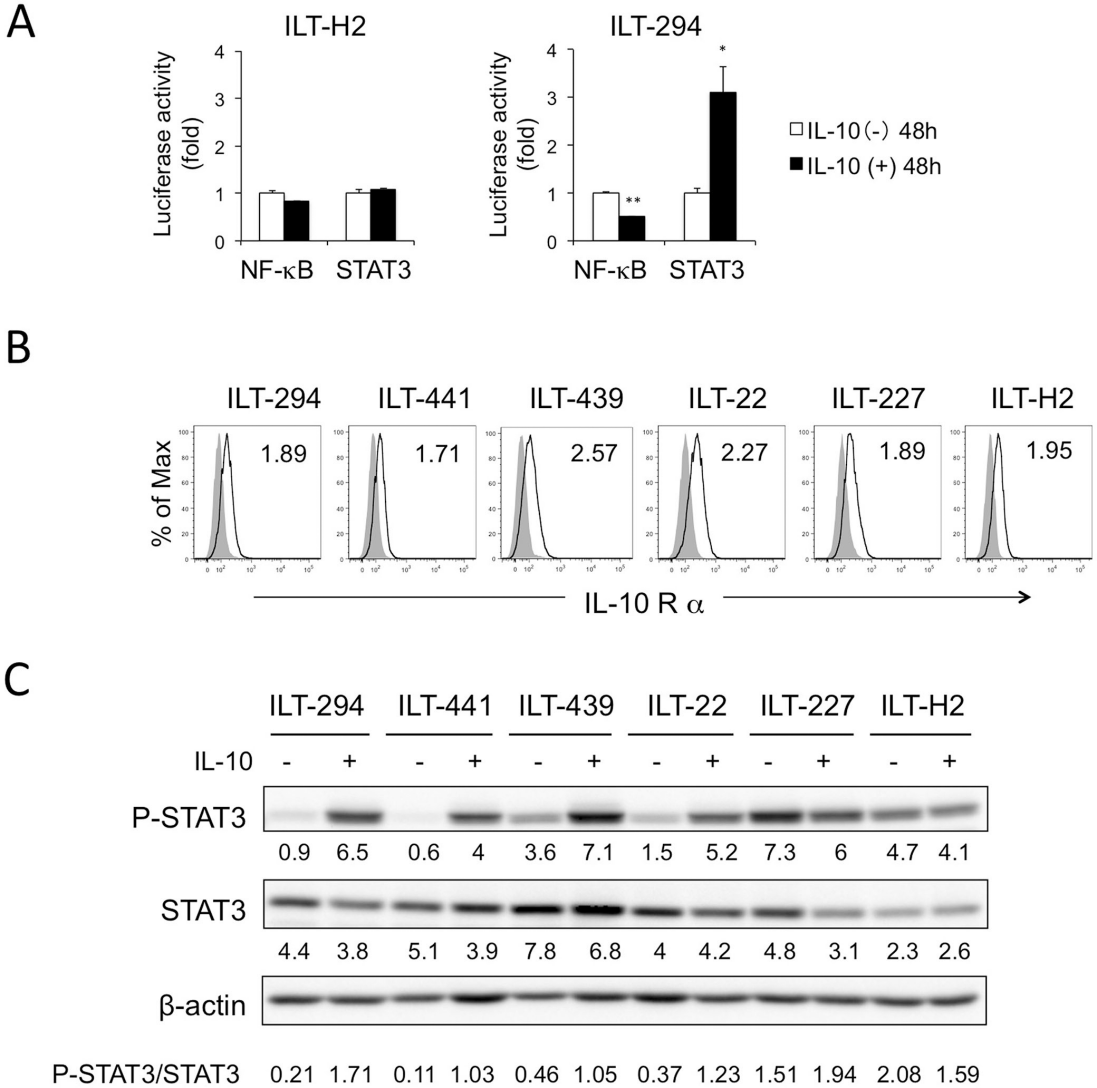
IL-10 induced STAT3-phosphorylation and mild suppression of NF-κB activity in ILTs. **A.** Luciferase activities in ILT-H2 and ILT-294 cells containing NF-κB-luc or STAT3-luc and TK-RL reporter genes were measured following incubation with (closed bar) or without (open bar) IL-10 (20 ng/ml) for 48 h. The NF-κB and STAT3 activities were standardized with TK promoter activities, and the relative values against the sample without IL-10 are presented as the mean and SD of duplicate samples. **B.** The surface IL-10Rα expression (open histogram) on various ILTs was assessed by flow cytometry. The numbers represent relative MFI of IL-10Rα compared with isotype controls (closed histogram). **C.** ILT cells were cultured with or without IL-10 for two (ILT-294 and -441) or one (ILT-439, -22, -227, and -H2) weeks, and cell lysates were probed with antibodies to phospho-STAT3 (p-STAT3), STAT3, and β-actin in an immunoblotting assay. The numbers under each band represent the relative values of intensity of the band against β-actin. The ratio of the value of p-STAT3 normalized against total STAT3 was also indicated in the bottom of the panel. Representative results of two independent experiments are shown. * *p*<0.05, ** *p*<0.01.

We next assessed signaling pathways involved in IL-10-mediated proliferation in ILTs. We focused on NF-κB and STAT3, because both transcription factors are constitutively activated in ATL cells and implicated in leukemogenesis [29, 43]. We used ILT-H2 and ILT-294 reporter cells that had been transduced with luciferase reporter genes driven by NF-κB and STAT3. Following IL-10-treatment, NF-κB activity was decreased, and STAT3 activity was markedly enhanced in ILT-294 cells (Fig 3A). In ILT-H2, the effects of IL-10-treatment were limited.

Since IL-10 activates the JAK-STAT signaling pathway through engagement of its cognate receptor [44], we assessed cell surface expression of IL-10 receptor α, and confirmed that the ILTs tested expressed IL-10Rα at similar levels (Fig 3B). We then evaluated the effects of rhIL-10 on STAT3 phosphorylation. Immunoblotting analysis revealed that phosphorylation of STAT3 was strongly enhanced in the presence of rhIL-10 in ILT-294, ILT-441, ILT-439, and ILT-22 (Fig 3C). In ILT-227 and ILT-H2, which spontaneously produced high levels of IL-10, STAT3 was strongly phosphorylated in the absence of exogenous rhIL-10, for which further IL-10-treatment showed little effect.

Nucleotide sequencing indicated that none of the ILTs tested had mutations in the hot spot regions (exon 20 and 21) of the *STAT3* gene [45, 46] (S4 Fig).

We also assessed NF-κB pathways by immunoblotting, and found a reduction in the amount of NF-κB p52, especially in ILT-294 cells, suggesting that IL-10 suppressed the non-canonical NF-κB pathway in this cell line (S5 Fig). A similar trend was observed in ILT-441 and ILT-22 cells but not in the other ILTs tested.

Thus, IL-10 strongly enhanced STAT3 phosphorylation with mild suppression of NF-κB signaling in the ILTs with low IL-10 production, especially those derived from HAM/TSP patients.

### STAT3 contributes to expression of *IL10*, *BIRC5*, *MYC* and *IRF4* in ILT cells

**Fig 4:**
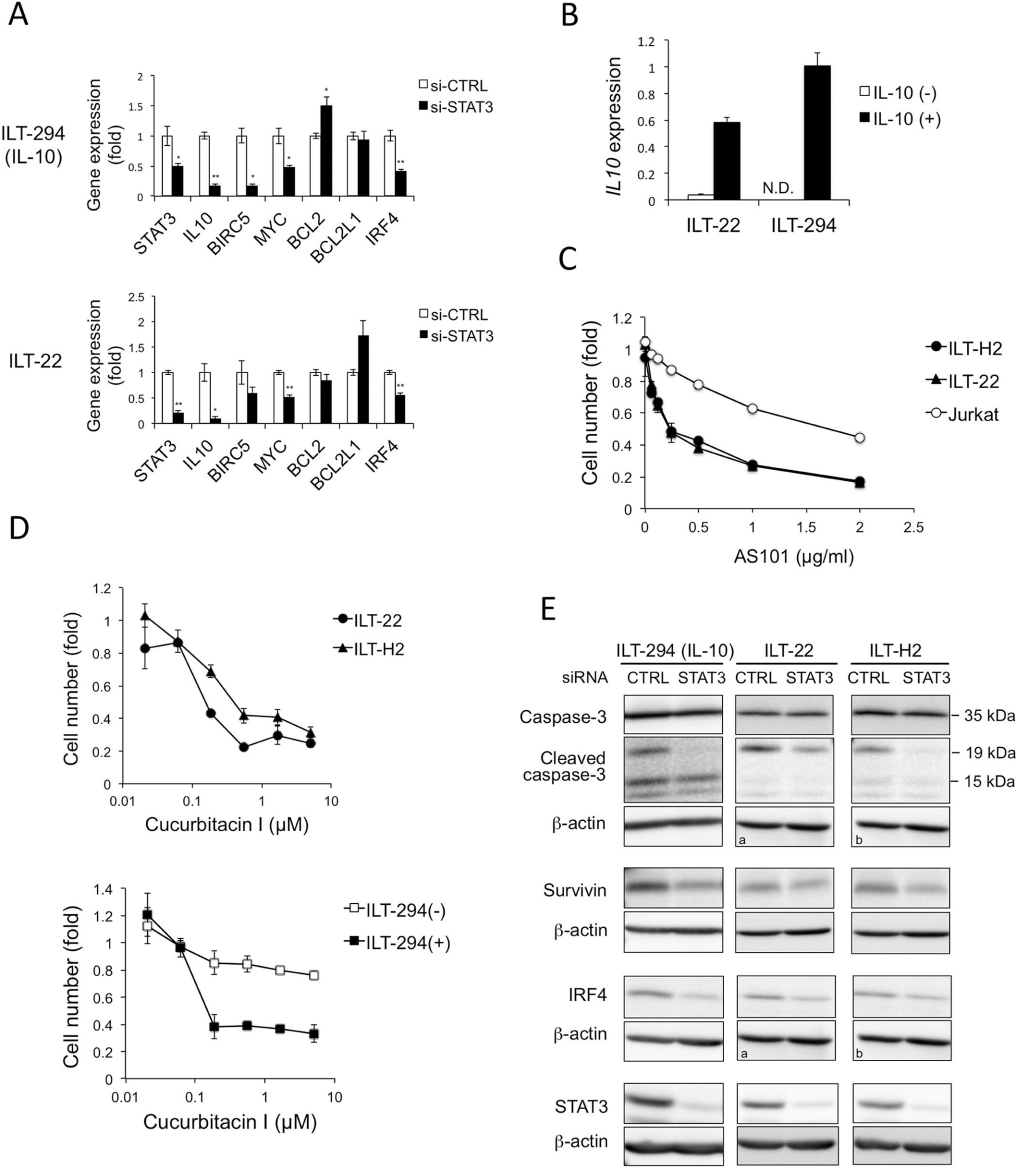
STAT3 knockdown inhibited expression of *IL10*, *BIRC5* (*survivin*), *MYC*, and *IRF4*. **A**. ILT cells were transfected with control siRNA (si-CTRL) (open bar) or si-STAT3 (black bar), and subjected to qRT-PCR 48 h after electroporation. Indicated gene expression levels were measured and standardized with *ACTB* (*β-actin*) mRNA levels in each sample. The relative values against the si-CTRL samples are presented as means and SD of duplicate samples. Representative results of two independent experiments are shown. * *p*<0.05, ** *p*<0.01. **B.** ILT-22 and ILT-294 cells cultured with (■) or without (□) rhIL-10 for at least 1 week, and *IL10* mRNA levels were measured. The normalized values against *ACTB* were indicated as means and SD of duplicate samples. N.D., not detected. **C.** ILT-H2 (●), ILT-22 (▲), and Jurkat (◯) cells were incubated with indicated concentrations of AS101 or vehicle control (0.13% ethanol) for 5 days and the viable cell number was assessed using Cell Counting Kit-8. Relative values against vehicle control were plotted as means and SD of duplicate samples. **D**. ILT-H2 (▲), ILT-22 (●), ILT-294 (+) (cultured with IL-10) (■), and ILT-294 (-) (cultured without IL-10) (□) cells were incubated with indicated concentrations of Cucurbitacin I or vehicle control (0.1% DMSO) for 2 days, and the cell number was evaluated by Cell Counting Kit-8. The relative values against vehicle controls indicate means and SD of duplicate samples. **E.** Cell lysates of ILTs were harvested 48 h (ILT-294, ILT-22) or 72 h (ILT-H2) after transfection with si-CTRL or si-STAT3, probed with antibodies to caspase-3, cleaved caspase-3, survivin, and IRF4 in immunoblotting assay, and presented together with the β-actin immunoblots of corresponding membranes. Approximate sizes of caspases are indicated. ^a^, ^b^, denote the same images for β-actin because the same membranes were used for the detection of caspases and IRF4, respectively. ILT-294 cells were cultured in the presence of IL-10 in **A** and **E**.

We next investigated the role of STAT3 in ILTs using siRNA for STAT3 (si-STAT3). In ILT-294 cells cultured in the presence of rhIL-10, si-STAT3 significantly suppressed mRNA levels of *STAT3*, *IL10*, *BIRC5* (*survivin*), *MYC*, and *IRF4*, but not *BCL2* or *BCL2L1* (*Bcl-xL*), when compared with control siRNA (Fig 4A, top). Similar results were obtained in ILT-22 cells (Fig 4A bottom).

The marked reduction in *IL10* expression by si-STAT3 suggested the presence of a positive feedback loop between STAT3 and IL-10. This idea was further supported by the finding that *IL10* expression was induced by treatment with rhIL-10 in the ILTs producing little or no IL-10 (Fig 4B). The role of the IL-10-STAT3 pathway in the cell growth in ATL-derived ILTs was further examined by using several inhibitors. AS101, a synthetic tellurium compound, has been reported to exhibit various immunomodulatory effects including IL-10 inhibition [47]. AS101 suppressed the growth of ILT-H2 and ILT-22 cells in 5 days of culture (Fig 4C). Knockdown of IL-10 also partly suppressed the cell growth in ILT-H2 but hardly in ILT-22 cells (S6 Fig). Moreover, a STAT3-inhibitor cucurbitacin I (JSI-124) [48] markedly inhibited the cell growth in ILT-H2 and ILT-22, as well as in ILT-294 cultured in the presence of rhIL-10 (Fig 4D). These findings indicate the importance of the IL-10-STAT3 pathway in the growth of ILT cells, to which the positive feedback loop may potentially contribute.

Following STAT3-knockdown, reduction of survivin, IRF4, and the 19 kDa band of cleaved caspase-3 was commonly observed in ILT-294 (with rhIL-10), ILT-22 and ILT-H2 cells (Fig 4E). Thus, STAT3 contributed to the IL-10-mediated or spontaneous cell growth in HAM/TSP-derived and ATL-derived ILTs, respectively, which was associated with induction of downstream molecules including survivin and IRF4.

### IRF4 expression is enhanced by IL-10 and contributes to cell growth and survivin induction in ILT cells

**Fig 5:**
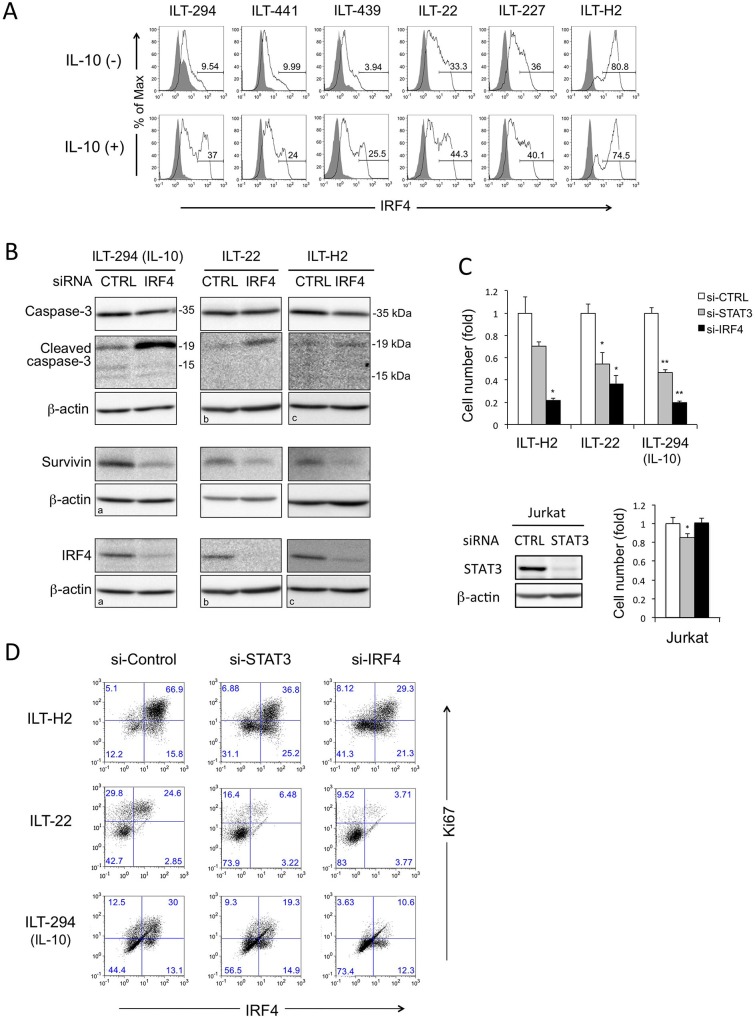
Critical roles of IRF4 in expansion of ILTs. **A.** Intracellular IRF4 expression (solid line) was analyzed in various ILT cells following incubation with or without IL-10 for 7–11 days. Closed histograms represent staining with control antibody. The numbers indicate the proportions (%) of cells with high IRF4 expression. **B.** ILT-294 (cultured with rhIL-10), ILT-22, and ILT-H2 cells were transfected with si-CTRL and si-IRF4, and the cell lysates were subjected to immunoblot assays for cleaved caspase-3, caspase-3, survivin, and β-actin 48 hours after electroporation. Approximate sizes of caspases are indicated. ^a^, ^b^, ^c^, denote the same images for β-actin because the same membranes were used, respectively. **C.** Indicated ILTs and Jurkat cells were transfected with si-CTRL, si-STAT3, or si-IRF4, and the total viable cell count were evaluated 3 days after electroporation by trypan blue exclusion test. The data represent the mean and SD of duplicate or triplicate samples. The immunoblot confirming the knockdown efficiency of STAT3 in Jurkat cells is also indicated. IRF4 was not detectable in Jurkat. **D.** Intracellular IRF4 and Ki67 expression of the ILTs prepared in C were analyzed by flow cytometry. ILT-294 cells were cultured in the presence of IL-10 in B, C, D. Numbers in D indicate the proportion (%) of cells in each quadrant. Similar results were obtained in two independent experiments. * *p*<0.05, ** *p*<0.01.

IRF4 expression has been reported previously in ATL cells [49, 50]. However, the relationship between IL-10 and IRF4 expression remains to be elucidated. We, therefore, examined intracellular IRF4 expression in ILTs by flow cytometry. The levels of IRF4 expression in HAM/TSP-derived ILTs tested were lower than in ATL-derived ILTs in the absence of rhIL-10 (Fig 5A). There were two populations with low and high IRF4 expression respectively, and the IRF4-high population was more common in ATL-derived ILTs. Exogenous rhIL-10 clearly enhanced IRF4 expression, especially in HAM/TSP-derived ILTs. A similar trend was also observed in ATL-derived ILTs, except for ILT-H2, which constitutively expressed the highest levels of IRF4 regardless of IL-10 treatment (Fig 5A).

Although several mutations in the exons 2 and 3 in the *IRF4* gene have been found in ATL [31], no mutations in those regions of the *IRF4* gene were found in the ILTs tested (S4 Fig).

To further investigate the role of IRF4 in the expansion of ILTs, we knocked down IRF4 using si-IRF4 in ILT-294 (cultured in the presence of rhIL-10), ILT-22 and ILT-H2 cells. Immunoblotting analysis confirmed that knockdown of IRF4 resulted in decreased survivin expression and increased the 19 kDa form of cleaved caspase-3 in these cells (Fig 5B). IRF4 knockdown mildly decreased *IL10* mRNA expression (S7 Fig).

The cell number was more efficiently decreased by IRF4 knockdown than STAT3 knockdown in ILT cells. The effects of STAT3 and IRF4 knockdown in Jurkat cells were limited (Fig 5C).

We also assessed the relationship between IRF4 and Ki67 by flow cytometry (Fig 5D). In ILT-H2 cells transfected with control siRNA, most of the cells strongly expressed both IRF4 and Ki67. Knockdown of STAT3 suppressed IRF4 expression, which was associated with decreased Ki67. Knockdown of IRF4 more clearly demonstrated that the decreased Ki67 expression was associated with IRF4 reduction. Similar results were obtained in ILT-22 cells and ILT-294 cells cultured in the presence of rhIL-10 (Fig 5D).

These results indicate that IL-10 enhances expression of IRF4 partly through STAT3, and IRF4 plays critical roles in the survival and proliferation of ILTs.

## Discussion

In the present study, by using patient-derived HTLV-1-infected T-cell lines that were not fully transformed, we demonstrated that the anti-inflammatory cytokine IL-10 and its downstream signaling play critical roles in the proliferation of HTLV-1-infected T lymphocytes. The proliferation speed of the ILTs varied widely among lines, even in the presence of rhIL-2, and appeared to be associated with their ability to produce IL-10. This was confirmed by the addition of rhIL-10 in the culture resulting in marked enhancement of cell growth, especially in ILTs derived from HAM/TSP patients. These findings imply that, while HTLV-1 infection alone may not be sufficient for proliferation of the infected cells, IL-10-dominant polarization of the infected cell itself or its microenvironment potentially contributes to proliferation of HTLV-1-infected cells. Lymphoproliferation is a characteristic of ATL, which is not associated with HAM/TSP. Therefore, IL-10-mediated signals might act as a switch toward leukemogenesis at the early stages of ATL development.

It has been reported previously that ATL cells frequently express IL-10 and the majority of acute ATL patients present with serum IL-10 levels higher than chronic ATL patients, asymptomatic HTLV-1 carriers and healthy donors, with serum IL-10 concentration increasing significantly with disease progression [22, 33]. Since IL-10 is an immunoregulatory cytokine suppressing inflammation and Th1 responses [36], the increased IL-10 levels in ATL patients have been implicated in the severe immunosuppression associated with ATL [33]. In contrast, no significant difference in serum IL-10 concentration was found between HAM/TSP patients and asymptomatic patients [34, 35]. Our present findings are consistent with these previous clinical observations, and further indicate the biological importance of IL-10 in proliferation of HTLV-1-infected cells. Notably, positive roles for IL-10 in the growth and survival of tumor cells have also been described in non-Hodgkin's lymphoma [51], Burkitt’s lymphoma [52] and non-small cell lung cancer [53].

In the present study, we demonstrated that both cell cycling and apoptosis occurred simultaneously in ILTs. IL-10 increased Ki67, cleaved caspase-3, and survivin expression (Fig 2). The presence of both Ki67 and cleaved caspase-3 has been reported previously in primary ATL cells [54]. Expression of survivin has also been reported in primary ATL cells, and high survivin expression is linked to low survival rates in ATL patients [40–42]. Given the fact that survivin is a member of the inhibitor of apoptosis family [55], the levels of apoptosis in ILTs might reflect the balance between pro- and anti-apoptotic machinery. It is speculated that ILTs continuously produce an apoptotic signal that takes over in the absence of IL-10. When these cells are exposed to IL-10, the apoptotic signal may be further enhanced, but apoptosis is blocked by survivin. The binding of survivin to p19 fragments of cleaved caspase-3 may block further cleavage resulting in accumulation of p19 fragments. Decreased p19 fragments of cleaved caspase-3 by STAT3 knockdown in ILTs confirmed that IL-10 induced this phenomenon through STAT3 (Fig 4E).

The signaling pathways downstream of IL-10 in ILTs involved STAT3 activation, which appeared to form a positive feedback loop for IL-10 production, as knockdown of STAT3 significantly decreased *IL10* gene expression (Figs 3C, 4A and 4B). This positive feedback potentially contributes to the overproduction of IL-10 in some ATL-derived ILTs. In IL-10-producing ATL-derived ILTs, however, the contribution of their own IL-10 to the cell growth seemed to be partial and varied among ILTs (S6 Fig), suggesting the additional presence of IL-10-independent mechanisms of cell growth in these cells. Nevertheless, the suppressive effects of AS101 and cucurbitacin I on the cell growth in ILTs imply the importance of the IL-10-STAT3 pathway in these cells, which might potentially be a therapeutic target (Fig 4C and 4D).

STAT3 was also involved in the expression of *BIRC5* (*survivin*), *MYC* and *IRF4* in ILTs, but not *BCL2* or *BCL2L1*, known target genes of STAT3 (Fig 4A). This might be partly because these cells are IL-2-dependent. The presence of rhIL-2 in the assay medium might have compensated for the effects of si-STAT3 on the genes that could be upregulated by IL-2.

A constitutively activated STAT3 pathway is frequently found in ATL, as well as other malignant diseases [43, 56]. Recent reports indicated that STAT3 mutations are frequently observed in ATL patients [45]. Once the cells acquire gain-of-function mutations of STAT3, IL-10 may be dispensable. In the ILT cells tested, however, no mutations were found in the hotspots of the STAT3 gene (S4 Fig), and IL-10 may still serve as a mediator to activate this pathway. Although IL-6 also activates STAT3 [57], the effects of IL-10 dominated in ILTs, as IL-10 strongly enhanced the growth and STAT3 phosphorylation of HAM/TSP-derived ILTs that produced high levels of IL-6 (Figs 1D, 1E and 3C).

ATL-derived ILT-H2 cells produced the highest levels of IL-10, and most strongly expressed Ki67 and IRF4 among the ILTs tested. Further treatment with rhIL-10 produced no change or sometimes slightly suppressed the cell growth (Fig 1E). Since this cell line constitutively exhibits high levels of phosphorylated STAT3, the additional activation might become toxic for the cells. It has been reported that excessive activation of the STAT3 pathway does not protect chronic lymphocytic leukemia cells from apoptosis [58].

Overexpression of IRF4 has been implicated in oncogenicity in multiple myeloma [59], the activated B cell-like subtype of diffuse large B cell lymphomas [60], primary effusion lymphoma [61] and peripheral T-cell lymphomas [62]. IRF4 directly enhances MYC, and MYC enhances IRF4, generating an autoregulatory circuit in multiple myeloma [63]. Besides chromosomal translocation, constitutive NF-κB activation causes overexpression of IRF4 [62]. HTLV-1-infected and ATL cells also express IRF4, and its contribution to enhanced cell growth by repressing DNA repair and pro-apoptotic genes has been suggested [50]. Although Tax can induce IRF4 expression, the mechanism of IRF4 expression in ATL cells remains controversial [49, 64, 65]. In the present study, IL-10 enhanced IRF4 expression, but mildly suppressed both NF-κB activity and Tax expression in HAM/TSP-derived ILT cells (Figs 5A, 1F and 3A and S5B Fig). This suggests that, although the constitutively active NF-κB activity in ILTs could partly contribute to IRF4 expression, IL-10 further augmented IRF4 expression through STAT3, independently of NF-κB or Tax. Interestingly, a previous study reported that constitutive IRF4 expression is exclusively observed in HTLV-1-transformed and ATL cells, but not in HTLV-1-infected cells from HAM/TSP patients [64].

Knockdown of IRF4 markedly reduced the cell number and survivin expression in ILTs, more efficiently than STAT3 knockdown (Fig 5B–5D), indicating a primary role of IRF4 in cell survival in ILTs. Knockdown of STAT3 did not fully suppress IRF4 expression, presumably because IRF4 is additionally regulated by NF-κB. It is also possible that STAT3 might indirectly enhance IRF4 by affecting other molecules. The different effect on the cleaved caspase-3 levels between si-STAT3 and si-IRF4 (Figs 4E and 5B) suggests the presence of simultaneous pro- and anti-apoptotic signals by STAT3, in which IRF4 predominantly contributes to anti-apoptotic signals. Intriguingly, recent reports indicated that IRF4 mutations are more common in aggressive ATL, while STAT3 mutations are characteristic of the indolent type of ATL [31, 66], suggesting unique roles of STAT3 in ATL leukemogenesis.

The reason for the variation in IL-10 production among ILTs is unknown. There were no clear differences in helper T-cell subsets among ILTs tested (S1 Fig). It might reflect differences in the host responses. Since our previous study suggested that HTLV-1 provirus-encoded antisense RNAs at the LTR region potentially activate PKR, leading to NF-κB activation [32], it is conceivable that such anti-viral intrinsic mechanisms could influence IL-10 induction. Notably, polymorphisms in the promoter region of IL-10 are thought to be associated with various diseases, the outcome of both HCV [67] and HIV-1 [68] infection. For HTLV-1 infection, the IL-10-592A/C SNP affects Tax-induced transcription and susceptibility to HAM/TSP, ratifying the importance of this cytokine in the disease outcome of HTLV-1 infected patients [69]. However, this SNP may not be applicable to the Brazilian HAM/TSP population [70]. Further mechanisms responsible for the difference in IL-10 responses in HTLV-1 infection remain to be clarified.

In conclusion, the anti-inflammatory cytokine IL-10 and its downstream signaling act as a switch for the proliferation of HTLV-1-infected cells through activation of the STAT3 and IRF4 pathways. The IL-10-dominant microenvironment may be a critical host factor allowing the HTLV-1-infected cells to proliferate in the early stages of HTLV-1 leukemogenesis, partly explaining the mechanism, which until now has been unexplained, how HTLV-1 causes lymphoproliferative and inflammatory diseases without disease-specific virus variation. The present findings will contribute not only to the understanding of the disease mechanisms, but also to prediction of the disease risks and prophylactic strategies against disease development in HTLV-1 infection.

## Materials and methods

### Ethics statement

This study was approved by the Medical Research Ethics Committee of Tokyo Medical and Dental University. IL-2-dependent HTLV-1-infected T-cell lines (ILTs) were established from the PBMCs of patients with HAM/TSP (#294, #439, #441) and ATL (#227) who donated their blood samples after providing written informed consent. We also used old established cell lines ILT-22 and ILT-H2 that had been similarly established from the PBMCs of ATL patients [71]. The original blood samples or PBMCs used for establishing ILTs were provided from the St. Marianna University School of Medicine (Kanagawa, Japan), Imamura Bun-in Hospital (Kagoshima, Japan) and Kumamoto University School of Medicine (Kumamoto, Japan) following anonymization.

### Cells

ILTs were established by long-term culture of PBMCs of HTLV-1-infected patients in the presence of rhIL-2 (Shionogi, Osaka, Japan) for at least 6 months, following stimulation with phytohemagglutinin or Dynabeads Human T-Expander CD3/CD28 (Invitrogen, Carlsbad, CA). ILT-22, ILT-227, and ILT-H2 are derived from patients with ATL, while ILT-294, ILT-439, and ILT-441 are derived from patients with HAM/TSP. ILTs were maintained in RPMI 1640 medium (Sigma-Aldrich, St. Louis, MO) containing 10% FBS (Sigma-Aldrich), 100 U/mL penicillin, 100 μg/mL streptomycin (Wako, Osaka, Japan), supplemented with 30–50 U/mL (ILT-22, -227, -H2) or 50–100 U/mL (ILT-294, -439 and -441) of rhIL-2. As the growth speed of HAM/TSP-derived ILTs was extremely slow, rhIL-10 (20 ng/mL, PeproTech, London, United Kingdom) was added to cultures to expand these cells, and then cultured in rhIL-2-containing medium without rhIL-10 at least for 1 week before use in most of the experiments. PBMCs from a healthy individual that had been activated by Dynabeads Human T-Expander CD3/CD28 (Invitrogen) were also used. HTLV-1-infected cell lines MT-2 [72] and TL-OmI [73], and HTLV-1-negative Jurkat [74] and MOLT4 [75] cells were cultured in RPMI 1640 medium containing 10% FBS.

### Antibodies and reagents

For flow cytometry, PE or FITC-labeled anti-human CD4, CD8a, PE-labeled anti-human CDw210 (IL-10 receptor α), FITC-labeled Annexin V (BD Biosciences, San Jose, CA), Alexa Fluor 488-labeled anti-human Ki-67 (Biolegend, San Diego, CA), PE-labeled anti-human IRF4 (eBioscience, San Diego, CA), and their isotype controls were used. To detect HTLV-1 Tax, Alexa Fluor 488-conjugated Lt-4 [76] and its isotype control (mouse IgG3) antibodies were used. For immunoblotting assays, antibodies specific for phospho-NF-κB p65 (Ser536), NF-κB p65, phospho-NF-κB p100 (Ser866/870), NF-κB p100/p52, cleaved caspase-3, caspase-3, survivin, phospho-STAT3 (Tyr705), STAT3 and IRF4 were purchased from Cell Signaling Technology (Beverly, MA, USA). Antibodies specific for α-tubulin and β-actin were obtained from Sigma-Aldrich (Buchs, Switzerland). AS101, the non-toxic tellurium IL-10-inhibitor (Tocris, Ellisville, MO) [47] and cucurbitacin I (JSI-124), a STAT3-inhibitor (Tocris) [48] were also used.

### Flow cytometry

For cell surface staining, cells were incubated with antibodies for 20 min on ice. For intracellular staining, cells were fixed and permeabilized using fixation/permeabilization buffers (eBioscience) according to the manufacturer’s instructions prior to incubation with antibodies. To detect intracellular Tax protein, cells were fixed with 20 μg/ml lysolecithin/1% paraformaldehyde for 5 min, and permeabilized with methanol for 15 min followed by treatment with 0.1% Triton-X for 5 min on ice, and then incubated with Alexa Fluor 488-labeled Lt-4 or isotype control antibodies. For multiplex bead-based immunoassay, culture supernatants were incubated with beads coated with antibodies to various cytokines using the LEGENDplex kit (Biolegend) following the manufacturer’s protocol. The stained cells and beads were analyzed on a MACSQuant Analyzer (Miltenyi Biotec, Bergish Gladbach, Germany), and the data were analyzed using FlowJo (Tree Star Inc., Ashland, OR) or LEGENDplex (Miltenyi Biotec) software, respectively.

### Immunoblotting

ILTs were lysed in RIPA buffer (50 mM Tris-HCl, pH 7.4, 150 mM NaCl, 1% Nonidet P-40, 0.1% SDS) containing protease and phosphatase inhibitor cocktail (Roche Diagnostics, Basel, Switzerland) and 1 mM PMSF. In some experiments, 10 μM of MG132 (Peptide Institute, Osaka, Japan) was added to the culture 3 h before harvest and also to the lysis buffer. Cleared cell lysates were denatured with sample buffer (Thermo Scientific, Waltham, MA) containing 2-mercaptoethanol, separated by SDS-PAGE and transferred to PVDF membrane (ATTO, Tokyo, Japan). The membranes were blocked with Block Ace (DS Pharma Biomedical, Tokyo, Japan) or BSA (Sigma-Aldrich) and incubated with the indicated primary antibodies followed by a secondary incubation with horseradish peroxidase-conjugated anti-rabbit IgG (Cell Signaling) or anti-mouse IgG (GE Healthcare, Pittsburg, PA) antibodies. Bands were visualized by chemiluminescent substrate Novex ECL (Invitrogen, Carlsbad, CA) and ImageQuant LAS 4000 mini (GE Healthcare). The images were analyzed using ImageJ software. Protein quantification was performed by using ImageQuant TL software (GE Healthcare).

### Luciferase assay

Lentivirus particles (Cignal Lenti Reporter Assay) (Qiagen, Duesseldorf, Germany) containing firefly luciferase reporter genes for NF-κB (NF-κB-luc), STAT3 (STAT3-luc) and renilla-luciferase reporter gene for thymidine kinase (TK-RL) were used for a transient reporter assay or establishment of stable reporter cell lines. For transient assays, ILTs were infected with a mixture of lentiviruses containing NF-κB-luc and TK-RL at a 2:1 ratio, and the luciferase activities were measured 4 days after infection. The stable ILT-H2 and ILT-294 reporter cells that have been established by infection with lentiviruses containing NF-κB-luc or STAT3-luc and TK-RL genes followed by puromycin selection were used for evaluating the effects of IL-10-treatment. Cells were lysed in Passive Lysis Buffer (Promega, Madison, WI) and the luciferase activities of the lysates were measured by a luminometer (Berthold, Bad Wildbad, Germany) using the Dual-Luciferase Reporter Assay System (Promega). Values were normalized using Renilla luciferase activity.

### RNA interference

10^6^–10^7^ cells were subjected to electroporation using a Neon Transfection System (Invitrogen, Eugene, OR) with 1 μM siRNA. The siRNA for STAT3 (si-STAT3: sense, 5’-CACAUGCCACUUUGGUGUUUCAUAA-3’), and control siRNA (si-CTRL 47: sense, 5’-AGGUAGUGUAAUCGCCUUG-3’) were obtained through custom services of Invitrogen. To knockdown IRF4 or IL10, three siRNAs targeting IRF4 (si-IRF4-HSS 105508, -HSS 105509, -HSS 105510) or IL-10 (si-IL10-HSS105365, -HSS105366, -HSS 179890) purchased from Invitrogen were used as a mixture, respectively. Cells were incubated for 48 h after electroporation, and then lysed in RIPA buffer for immunoblotting or in ISOGEN (Nippon Gene, Tokyo, Japan) for RNA extraction. For flow cytometry, cells were harvested 72 h after electroporation.

### Primers

The nucleotide sequences of the primers used for RT-PCR, proviral load measurement [17], and DNA sequencing of the mutation hot spots of STAT3 [46] and IRF4 [77] genes are shown in S1 Table. The NCBI Primer-Blast Tool and qPrimerDepot database were used for designing primers.

### Quantitative RT-PCR

RNA extracted from cells was DNase treated (Ambion, Austin, TX) and reverse transcribed using First Strand cDNA Synthesis Kit with oligo(dT)20 (TOYOBO, Osaka, Japan). The resulting cDNA was then used as a template for quantitative RT-PCR using THUNDERBIRD SYBR qPCR Mix (TOYOBO) and a LightCycler 2.0 (Roche). Quantified mRNAs were normalized to the *ACTB or GAPDH* mRNA level.

### Proviral load

DNA was extracted from ILT cells by using DNeasy Blood & Tissue kits (QIAGEN, Courtaboeuf, France) and subjected to quantitative PCR with HTLV-1 Tax-specific primers. The copy number per cell was calculated based on the *Beta-globin* copy number and further normalized against HTLV-1 proviral number of TL-OmI, which has been reported as 1.8 copies per cell [78]. The primers used are listed in S1 Table [17].

### Statistical analysis

Statistical significance was tested by a two-tailed unpaired *t* test, and the difference between groups considered significant at *p* < 0.05.

## Supporting information

S1 Table Primers used for PCR and RT-PCR. (TIF)

S1 Fig Expression of *TBX21*, *GATA3*, *RORC* and *FOXP3* in ILT cells. Expression of *TBX21*, *GATA3*, *RORC* and *FOXP3* mRNA in ILT cells and the PBMCs of a seronegative donor that had been stimulated with CD3/CD28 dynabeads for 3 days (SN-PBMC) were determined by qRT-PCR. The mRNA expression levels were normalized to *GAPDH* level, and the relative values against the SN-PBMC are presented as means and SD of duplicate or triplicate reactions. Representative results of two independent experiments are shown. N.D., not detected.
(TIF)

S2 Fig Effect of IL-10 on apoptosis in ILTs. Representative flow cytometry images of the data shown in Fig 2B. ILTs were cultured with or without IL-10 for 7–11 days, and stained with Annexin V. The values indicate apoptotic cells (%).
(TIF)

S3 Fig Effects of IL-10 on cleavage of caspase 3 in ILTs. ILTs cultured with or without IL-10 were subjected to immunoblot assays probed with antibodies to caspase-3, cleaved caspase-3, and β-actin. The results of a similar experiment with MG132-treatment is shown in Fig 2C.
(TIF)

S4 Fig Absence of mutations in the hotspots of the *STAT3* and *IRF4* genes in ILTs. Genomic DNA was extracted from the ILTs and subjected to PCR amplification of specific exons, followed by direct sequencing of PCR products. Sequence comparison between ILTs and wild type *STAT3* (NCBI Reference Sequence NG_007370.1) (A) and *IRF4* (NCBI Reference Sequence NG_027728.1) (B) genes are shown, with the mutation hotspots shaded [31, 45, 46]. Numbers indicate the position (bp) of the nucleotide within each exon.
(TIF)

S5 Fig Effects of IL-10 treatment on the NF-κB pathway in ILTs. NF-κB proteins in ILT cells cultured in the presence or absence of rhIL-10 were analyzed by immunoblotting assays following treatment with or without MG132 (10 μM) for the last 3 h of culture. Cell lysates were probed with antibodies to phospho-NF-κB p65 (p-p65) and NF-κB p65 (A), as well as phospho-NF-κB p100 (p-p100) and NF-κB p100/p52 (B). For loading controls, α-tubulin (ILT-294) or β-actin (ILT-441, -22, -227, -H2) were detected.
(TIF)

S6 Fig Effects of IL-10 knockdown on the cell growth in ATL-derived ILTs. **A.** ILT-22 and ILT-H2 cells were transfected with control (si-CTRL) and IL-10-specific (si-IL10) si-RNA, and the *IL10* mRNA levels (left) and the cell number (right) were evaluated by RT-PCR and trypan blue exclusion assay, respectively, 3 days after electroporation. The relative values against si-CTRL were indicated as means and SD of duplicate samples. **B.** ILT-22 and ILT-H2 cells were similarly transfected with si-CTRL or si-IL10, following pre-culture with IL-2-free medium for 24h. The cells were then cultured in IL-2-containing medium for 3 (ILT-22) and 4 (ILT-H2) days, and the cell number was evaluated as indicated above.
(TIF)

S7 Fig Mild suppressive effects of IRF4 knockdown on *IL10* expression in ILTs. ILT-H2 cells were transfected with si-CTRL or si-IRF4 and the *IL10* mRNA expression was evaluated 48 h after electroporation. The relative value against si-CTRL was indicated as the mean and SD of duplicate samples.
(TIF)
