# Supplementary figures and images for: IL-10-mediated signals act as a switch for lymphoproliferation in Human T-cell leukemia virus type-1 infection by activating the STAT3 and IRF4 pathways
Source: PLoS Pathog. 2017 Sep 14;13(9):e1006597. doi: 10.1371/journal.ppat.1006597 (PMC5614654; doi:10.1371/journal.ppat.1006597)

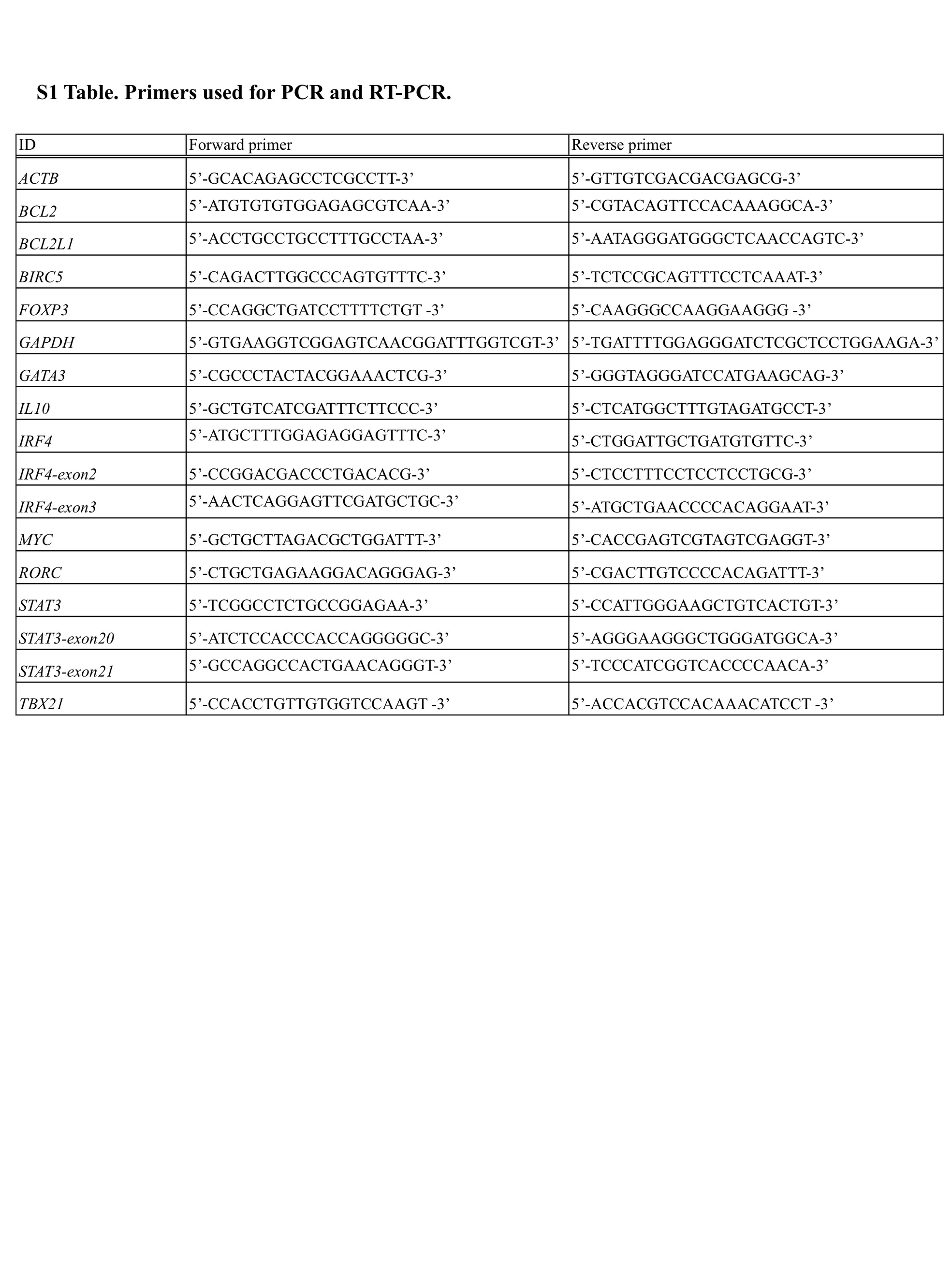

Supplement: S1 Table — (TIF) [file ppat.1006597.s001.tif]

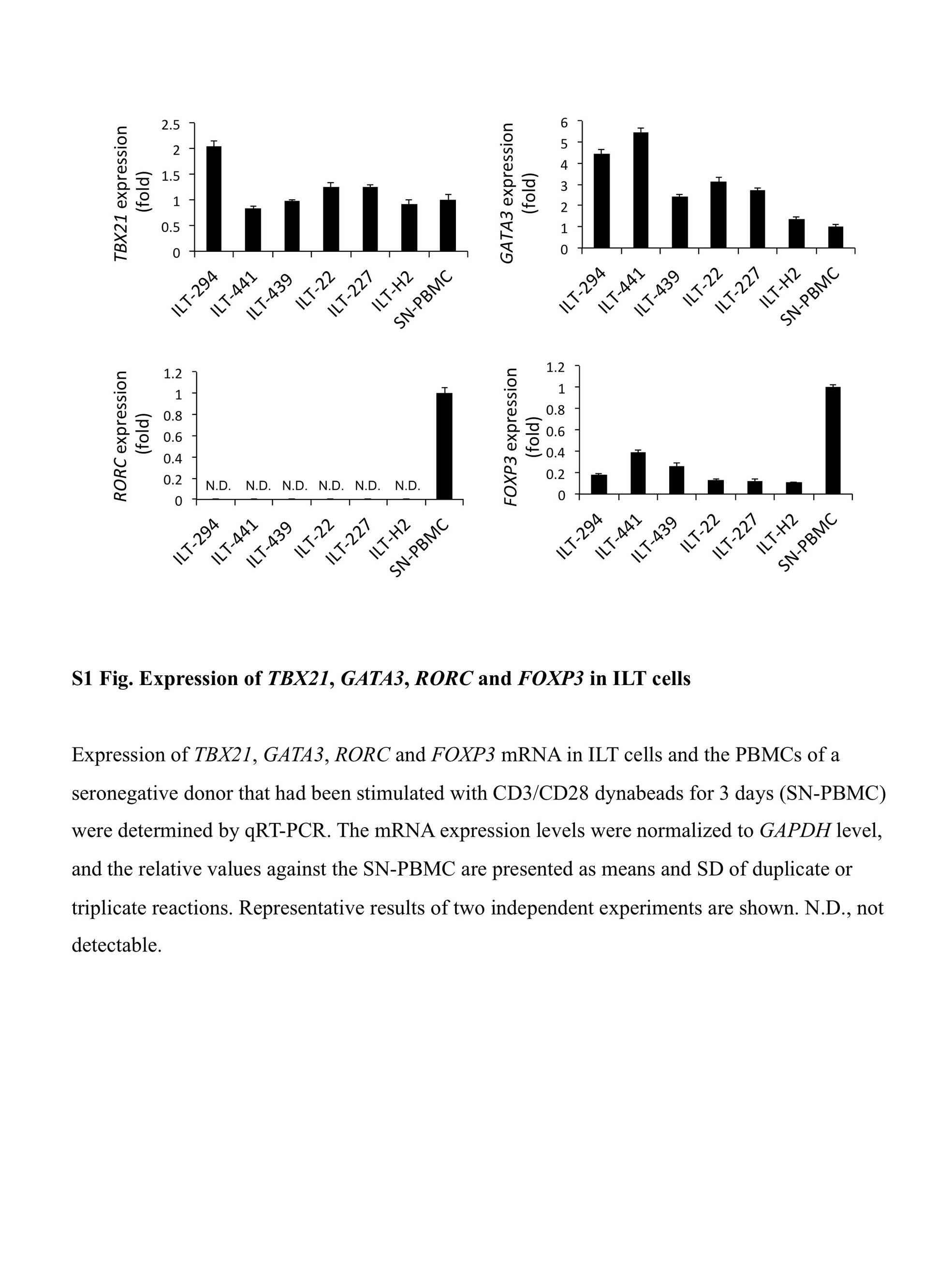

Supplement: S1 Fig — Expression of TBX21, GATA3, RORC and FOXP3 mRNA in ILT cells and the PBMCs of a seronegative donor that had been stimulated with CD3/CD28 dynabeads for 3 days (SN-PBMC) were determined by qRT-PCR. The mRNA expression levels were normalized to GAPDH level, and the relative values against the SN-PBMC are presented as means and SD of duplicate or triplicate reactions. Representative results of two independent experiments are shown. N.D., not detected. (TIF) [file ppat.1006597.s002.tif]

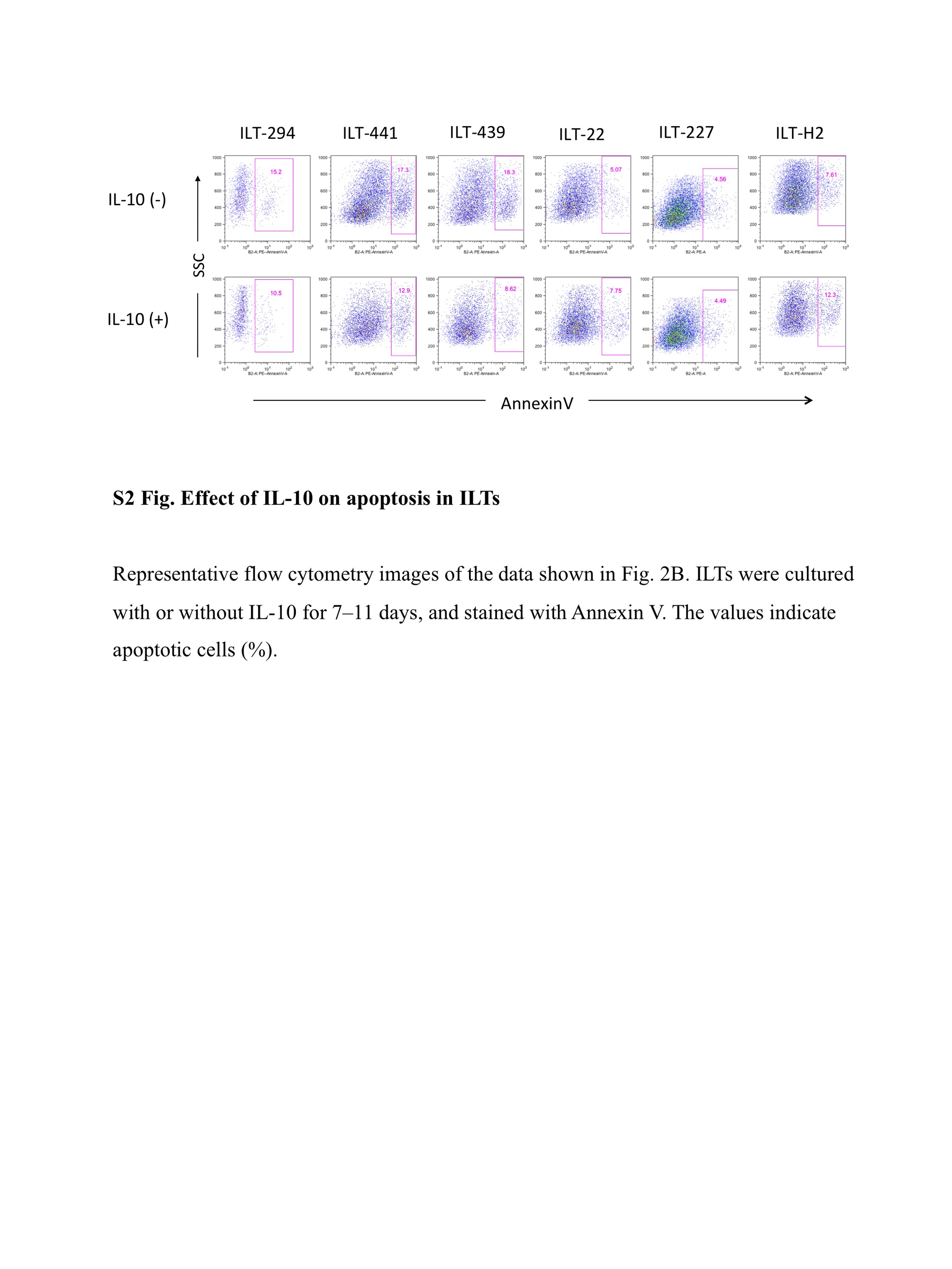

Supplement: S2 Fig — Representative flow cytometry images of the data shown in Fig 2B. ILTs were cultured with or without IL-10 for 7–11 days, and stained with Annexin V. The values indicate apoptotic cells (%). (TIF) [file ppat.1006597.s003.tif]

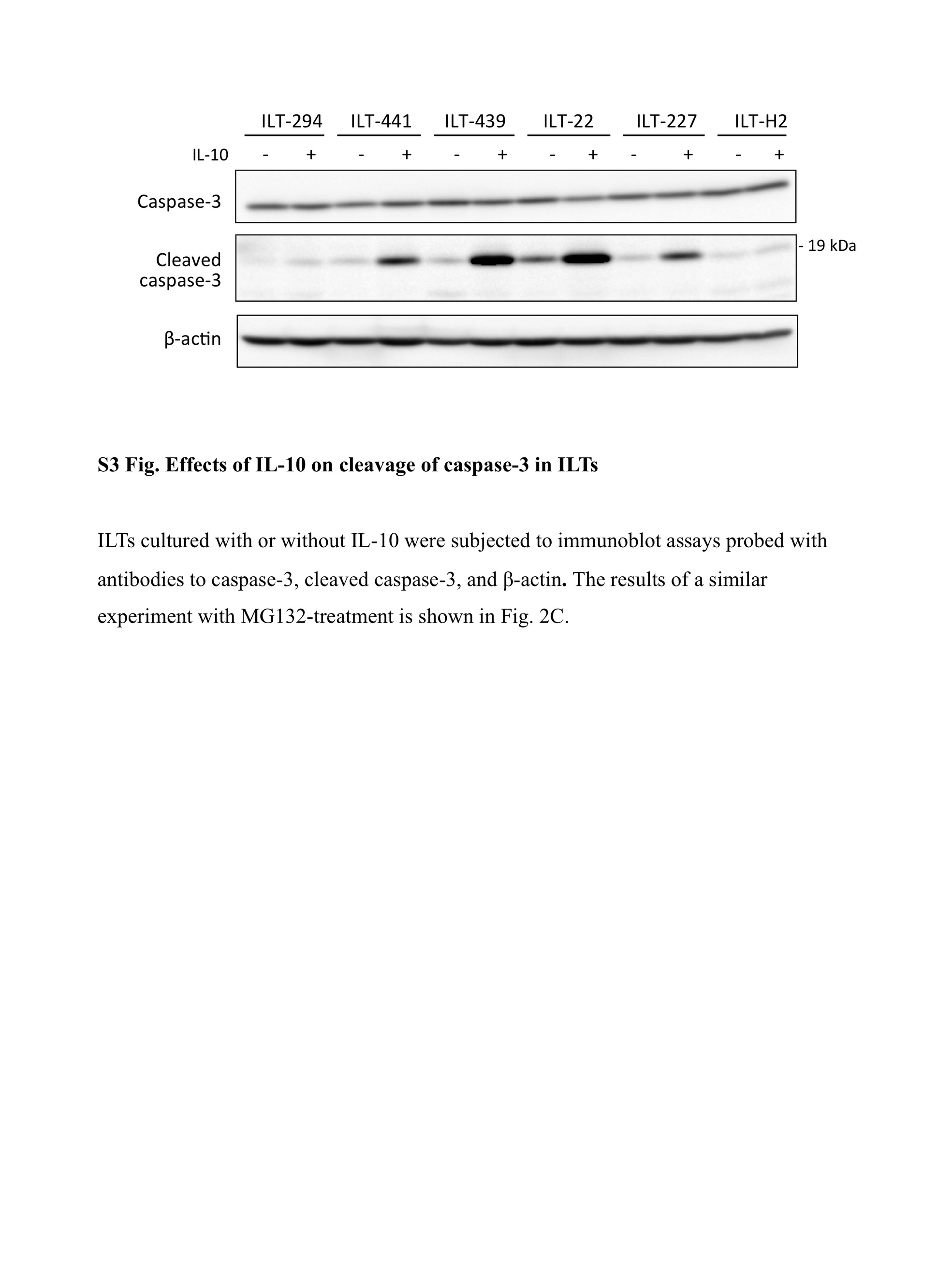

Supplement: S3 Fig — ILTs cultured with or without IL-10 were subjected to immunoblot assays probed with antibodies to caspase-3, cleaved caspase-3, and β-actin. The results of a similar experiment with MG132-treatment is shown in Fig 2C. (TIF) [file ppat.1006597.s004.tif]

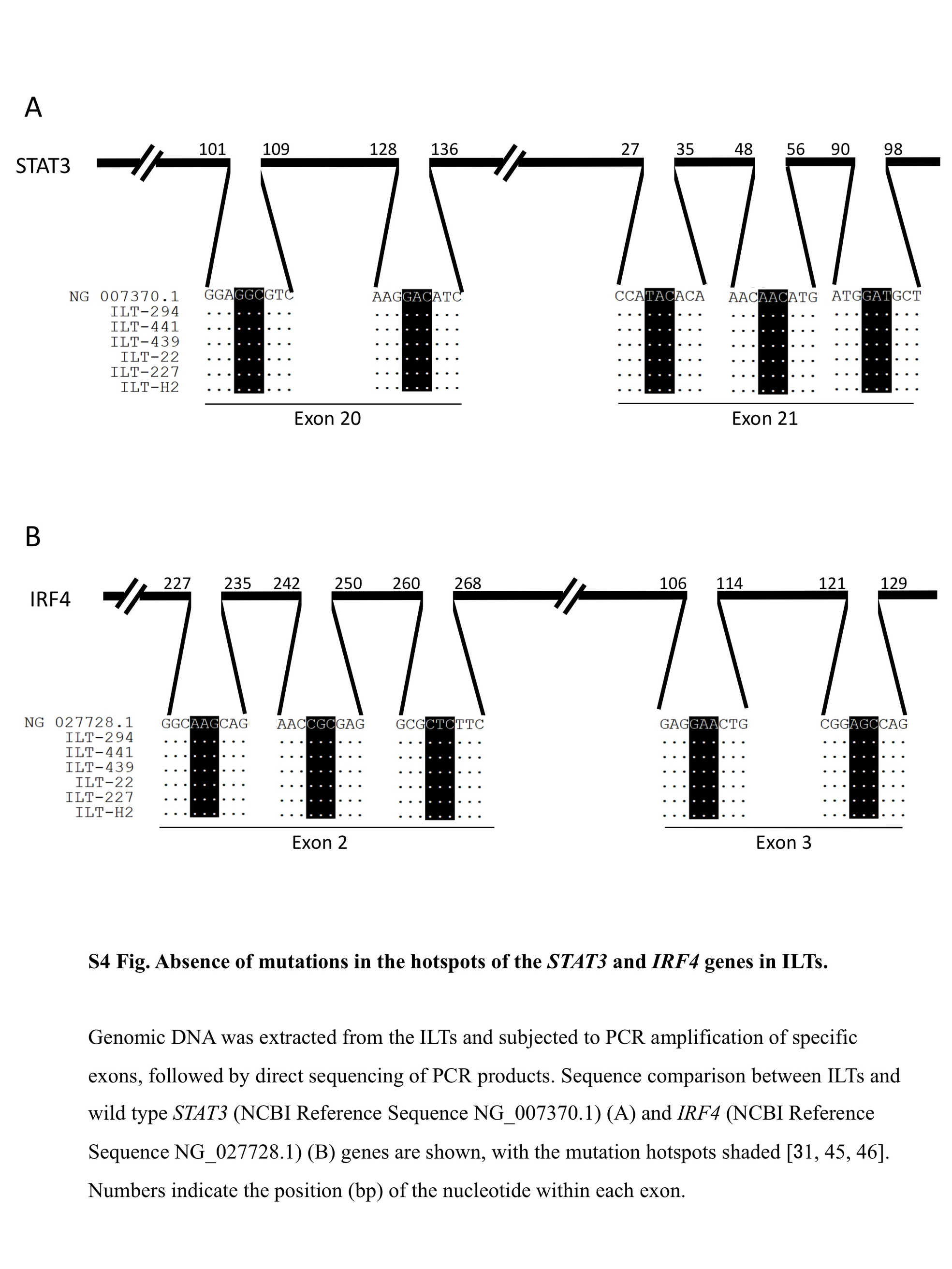

Supplement: S4 Fig — Genomic DNA was extracted from the ILTs and subjected to PCR amplification of specific exons, followed by direct sequencing of PCR products. Sequence comparison between ILTs and wild type STAT3 (NCBI Reference Sequence NG_007370.1) (A) and IRF4 (NCBI Reference Sequence NG_027728.1) (B) genes are shown, with the mutation hotspots shaded [31, 45, 46]. Numbers indicate the position (bp) of the nucleotide within each exon. (TIF) [file ppat.1006597.s005.tif]

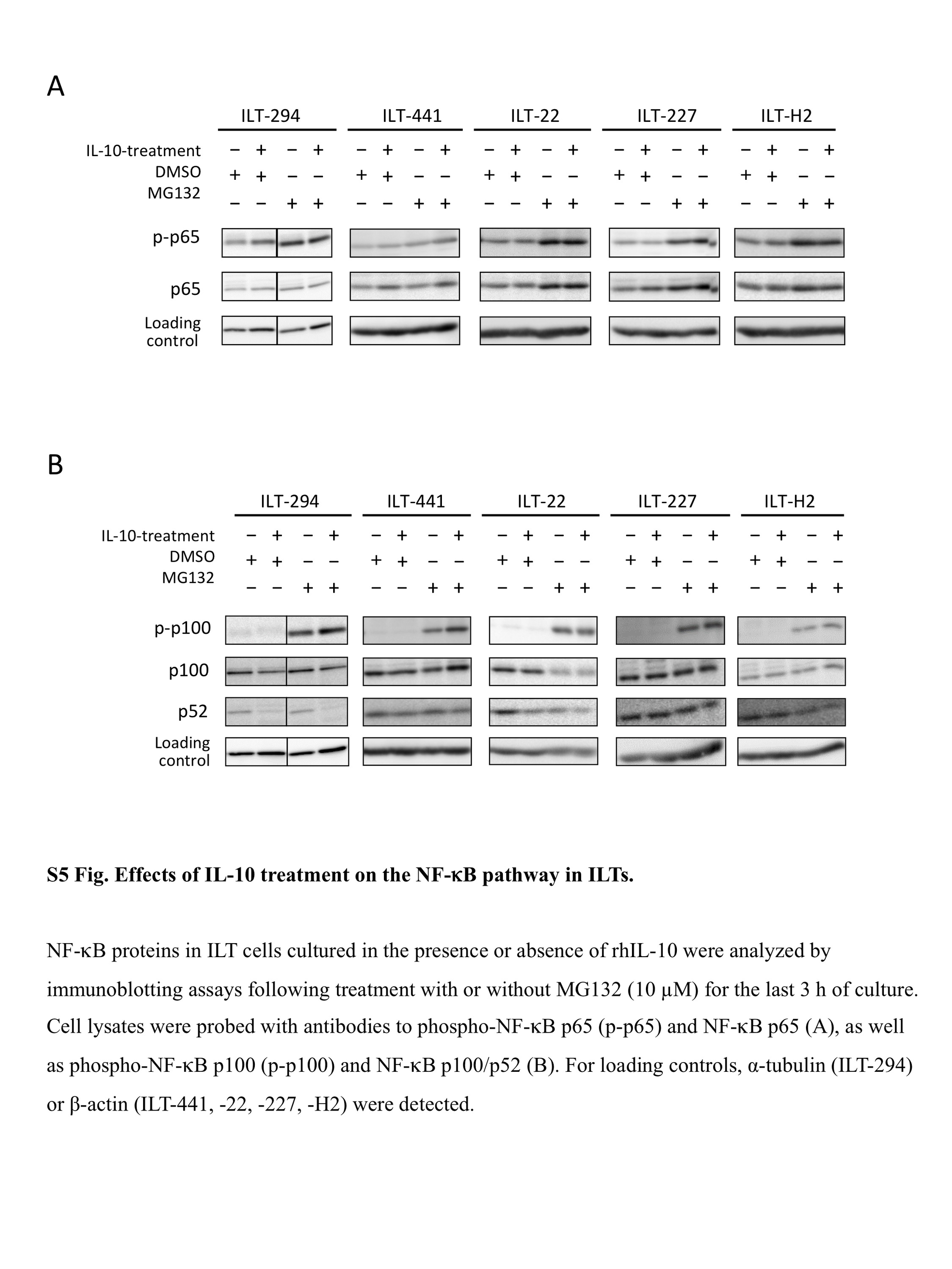

Supplement: S5 Fig — NF-κB proteins in ILT cells cultured in the presence or absence of rhIL-10 were analyzed by immunoblotting assays following treatment with or without MG132 (10 μM) for the last 3 h of culture. Cell lysates were probed with antibodies to phospho-NF-κB p65 (p-p65) and NF-κB p65 (A), as well as phospho-NF-κB p100 (p-p100) and NF-κB p100/p52 (B). For loading controls, α-tubulin (ILT-294) or β-actin (ILT-441, -22, -227, -H2) were detected. (TIF) [file ppat.1006597.s006.tif]

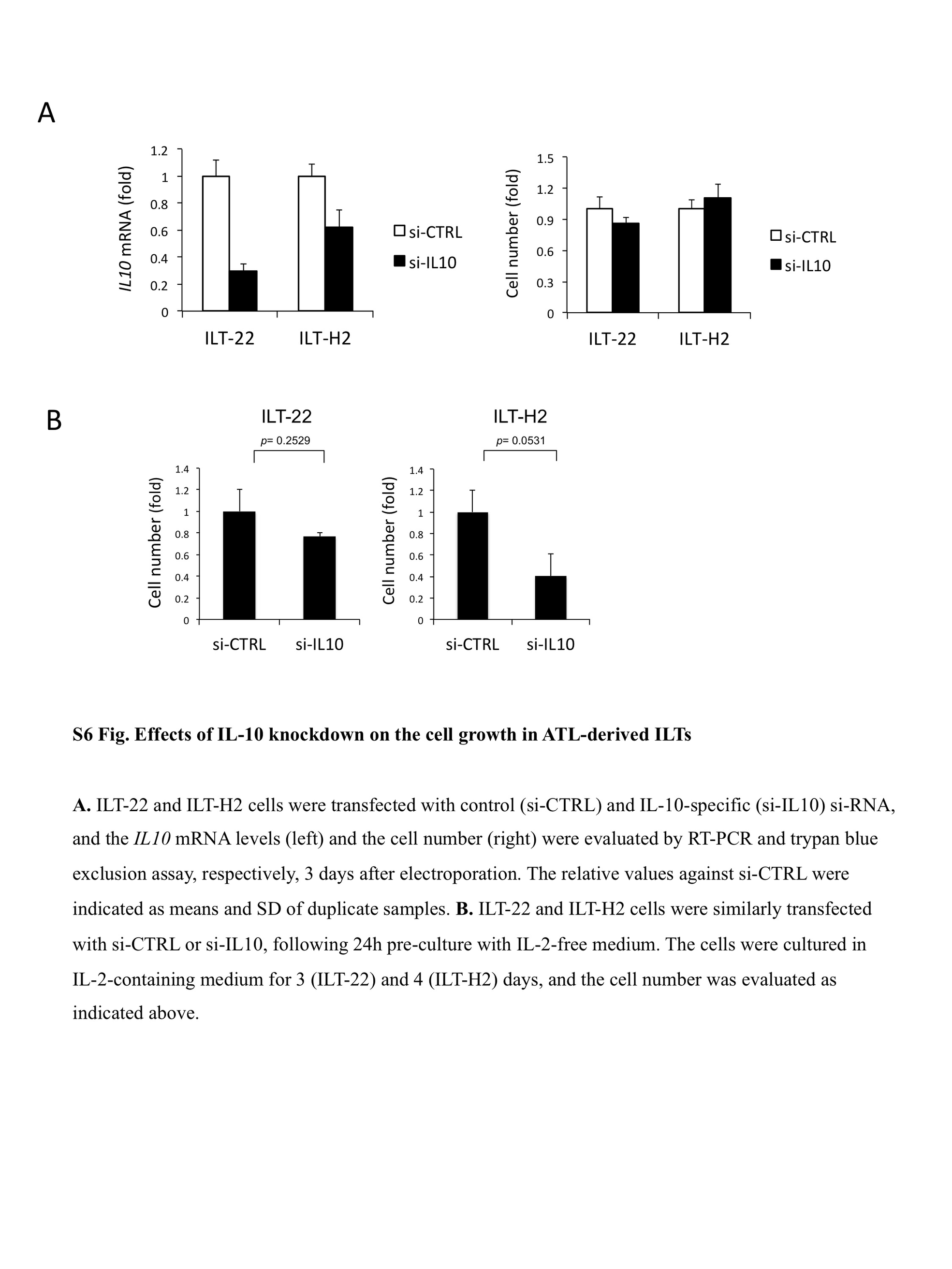

Supplement: S6 Fig — A. ILT-22 and ILT-H2 cells were transfected with control (si-CTRL) and IL-10-specific (si-IL10) si-RNA, and the IL10 mRNA levels (left) and the cell number (right) were evaluated by RT-PCR and trypan blue exclusion assay, respectively, 3 days after electroporation. The relative values against si-CTRL were indicated as means and SD of duplicate samples. B. ILT-22 and ILT-H2 cells were similarly transfected with si-CTRL or si-IL10, following pre-culture with IL-2-free medium for 24h. The cells were then cultured in IL-2-containing medium for 3 (ILT-22) and 4 (ILT-H2) days, and the cell number was evaluated as indicated above. (TIF) [file ppat.1006597.s007.tif]

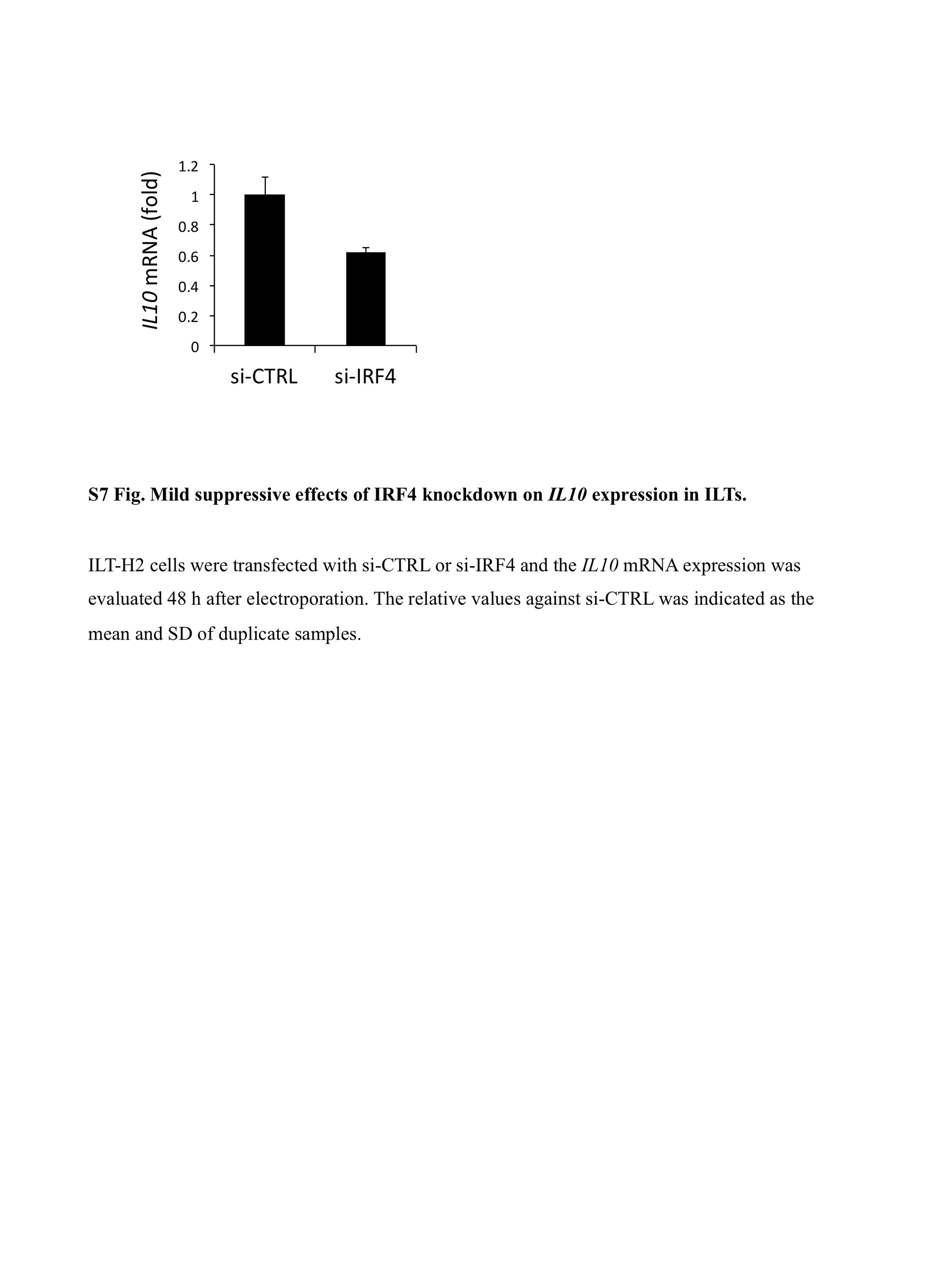

Supplement: S7 Fig — ILT-H2 cells were transfected with si-CTRL or si-IRF4 and the IL10 mRNA expression was evaluated 48 h after electroporation. The relative value against si-CTRL was indicated as the mean and SD of duplicate samples. (TIF) [file ppat.1006597.s008.tif]
